# PAC-UF Process Improving Surface Water Treatment: PAC Effects and Membrane Fouling Mechanism

**DOI:** 10.3390/membranes12050487

**Published:** 2022-04-29

**Authors:** Tian Li, Hongjian Yu, Jing Tian, Junxia Liu, Tonghao Yuan, Shaoze Xiao, Huaqiang Chu, Bingzhi Dong

**Affiliations:** 1 Key Laboratory of Yangtze River Water Environment, Ministry of Education, College of Environmental Science and Engineering, Tongji University, Shanghai 200092, China; litian001@tongji.edu.cn (T.L.); dbz77@tongji.edu.cn (B.D.); 2 State Key Laboratory of Pollution Control and Resource Reuse, College of Environmental Science and Engineering, Tongji University, Shanghai 200092, China; 1853855@tongji.edu.cn (H.Y.); tianjing1605@163.com (J.T.); 1952483@tongji.edu.cn (T.Y.); shaoze_xiao@163.com (S.X.); 3 Shanghai Institute of Pollution Control and Ecological Security, Tongji University, Shanghai 200092, China; 4 School of Civil and Transportation Engineering, Guangdong University of Technology, Guangzhou 510006, China

**Keywords:** powdered activated carbon, ultrafiltration, particle size, cleaning, fouling

## Abstract

In this study, the water purification effect and membrane fouling mechanism of two powdered activated carbons (L carbon and S carbon) enhancing Polyvinylidene Fluoride (PVDF) ultrafiltration (UF) membranes for surface water treatment were investigated. The results indicated that PAC could effectively enhance membrane filtration performance. With PAC addition, organic removal was greatly enhanced compared with direct UF filtration, especially for small molecules, i.e., the S-UF had an additional 25% removal ratio of micro-molecule organics than the direct UF. The S carbon with the larger particle size and lower specific surface area exhibited superior performance to control membrane fouling, with an operation duration of S-UF double than the direct UF. Therefore, the particle size and pore structure of carbon are the two key parameters that are essential during the PAC-UF process. After filtration, acid and alkaline cleaning of UF was conducted, and it was found that irreversible fouling contributed the most to total filtration resistance, while the unrecoverable irreversible resistance ratio with acid cleaning was greater than that with alkaline cleaning. With PAC, irreversible UF fouling could be relieved, and thus, the running time could be extended. In addition, the membrane foulant elution was analyzed, and it was found to be mainly composed of small and medium molecular organic substances, with 12% to 21% more polysaccharides than proteins. Finally, the hydrophilicity of the elution was examined, and it was observed that alkaline cleaning mainly eluted large, medium, and small molecules of hydrophilic and hydrophobic organic matter, while acid cleaning mainly eluted small molecules of hydrophilic organic matter.

## 1. Introduction

Powdered activated carbon (PAC) adsorption is a powerful and easily adjustable technology due to the effective removal of many contaminants [1,2,3]. The process of PAC adsorption to remove aquatic contaminants from natural water can be described with a three-step transition, i.e., from water to carbon, then to the carbon surface, and finally to the binding sites [4].

Membrane filtration has been identified as a safe barrier to microorganisms, suspended particles, and colloids through size exclusion, and ultrafiltration (UF) can be such a way to remove contaminants from surface water for its compactness, easy automation, and high removal ratio of turbidity [5,6,7]. However, membrane fouling is an urgent problem in membrane technology applications for water and wastewater treatment [6,8,9,10]. Hybrid PAC-microfiltration/ultrafiltration (MF/UF) has become an emerging water treatment technology for the thermos dynamically unstable surface and widely commercial availability of PAC [1,11]. Meanwhile, by reducing acid-base interaction energy, PAC can also control membrane fouling [12]. In the PAC-UF/MF process, PAC can effectively relieve membrane fouling since PAC can reduce the natural organic matter (NOM) deposition on the membrane surface or in the pores, thus extending the membrane filtration cycle and even enhancing organic matter adsorption and removal more than two-fold [13,14]. However, there are still some contradictory results regarding PAC influence on membrane filtration performance. Some researchers found that the higher the permeate flux, the longer the filtration duration or the lower the frequency of chemical washing [15,16]. In contrast, others reported that PAC-UF exhibited a similar flux to UF, and even flux decline occurred [17,18,19]. In the study of Shao et al., it was found that external fouling was caused by the deposition of biological PAC on the membrane surface, and during the operation of PAC-MBR, low flux and effective physical cleaning protocols were needed [20].

The effect of PAC on membrane fouling can be attributed to the membrane characteristics of hydrophobicity. Some authors have reported that PAC could reduce the flux decline of hydrophilic membranes to some extent, but it has almost no influence on the flux of the hydrophobic membranes [21,22]. Meanwhile, PAC has a mechanical effect on the performance of the membrane, as it is not usually separated in front of the membrane process. In other words, they influence the membrane process through the adsorption of solutes as well as through their properties as geometric bodies [18].

Since the different types of PAC with various properties can be responsible for the operation results of the PAC-UF system, the objective of this study is to investigate the operation process and the fouling control effect of two different types of PAC with different particle sizes and pore structures on the UF filtration process, especially for the long duration of combined operations for surface water treatment. In addition, the UF membrane cleaning effect associated with membrane resistance and elution characteristics is also analyzed systematically, such that the mechanism of PAC properties on UF performance could be elucidated. Before the detailed results are reported, the materials and methodologies are first reported in the following section.

## 2. Materials and Methodologies

### 2.1. PAC

Two commercial powdered activated carbons were used in this study, i.e., Li yuan carbon (L carbon, Fujian Yuanli Active Carbon Co., Ltd., Nanping, China) and Su carbon (S carbon, Suzhou Water Supply Co., Ltd., Suzhou, China). They were added during filtration to keep the mixed liquor suspended solids (MLSS) in the mixing reactor at about 4 g/L. At the beginning of the operation, an appropriate amount of glucose was artificially added to promote the growth of microorganisms. After each sampling, carbon was replenished to maintain the stability of the MLSS.

The two carbon size distributions were similar, mainly between 10 and 100 μm (Figure S1 and Table S1 in Supplementary Materials). The average particle size of the L carbon was smaller. The pore volume of the two types of carbons varies in different pore size ranges, but the pore volume of L carbon was generally larger than that of S carbon, especially in the pore size range of 0–15 nm (Figure S2). The specific surface area of the activated carbons was composed of micropore (d < 2.0 nm) and primary mesopore (d < 5.0 nm) (Figure S3), with L carbon much larger than S carbon. The content of acidic oxygen-containing functional groups on the surface of L carbon was higher, while the content of basic oxygen-containing functional groups on the surface of S carbon was larger (Table S2).

### 2.2. Source Water

The raw water was collected from Sanhaowu Lake at Tongji University, Shanghai, China. The pH, turbidity, DOC, UV_254_, NH_4_-N values of the water sample were 7.90 ± 0.06, 2.83 ± 1.18 NTU, 3.27 ± 0.63 mg/L, 0.068 ± 0.009 cm^−1^ and 0.50 ± 0.38 mg/L (*n* = 8), respectively.

### 2.3. PAC-UF Experimental Setup

Two reactors, A and B, were used in the experiment. Rector A was a direct UF filtration setup, and Reactor B was an activated carbon-reinforced setup. Both adopted immersed hollow fiber membrane filtration, with the setup diagrams shown in Figure S4.

The Polyvinylidene Fluoride (PVDF) ultrafiltration membrane used in this study has a membrane pore size of 0.05 μm and an effective surface area of about 0.12 m^2^ in a single module. The membrane flux was set to 30 L/(m^2^·h). The device adopted a negative suction pressure for filtration. After the initial sinking, the raw water was injected into the mixing tank with the feed water pump, and the liquid level of the mixing tank was controlled with a high-level floating ball valve. The mixing tank was connected with the submerged UF membrane tank, and the total volume of the mixed liquid of the two tanks was always maintained at about 16 L. Perforated pipe aerators were installed at the bottom of the mixing tank and the UF membrane tank. The membrane permeate was stored in a clean water tank, and excess water was discharged through the overflow pipe.

### 2.4. Membrane Cleaning Protocol

The contaminated membrane module was disassembled from the reactor after the experiment. In order to distinguish the source and size of reversible pollution from irreversible pollution, the physical and chemical cleaning of the contaminated membrane was carried out. In the chemical cleaning, HCl and NaOH solutions with the same mass concentration of 0.2 wt% were adopted. The cleaning setup diagram is shown in Figure S5.

The two cleaning tanks were connected in the middle, and a quantitative amount of ultrapure water (physical cleaning) or chemical cleaning liquid (chemical cleaning) was poured into the tank; after the fouled membrane module was fixed, water was supplied from pipeline 7 and discharged from pipeline 5. When the cross-flow cleaning mode was turned on, the cleaning liquid was supplied with water from pipeline 6; when the backwash mode was turned on, the water was supplied from pipeline 8. The cleaning cycle mode was automatically controlled with the Programmable Logic Controller (PLC). After these operations, the cleaning solution and membrane module were collected for subsequent analysis.

### 2.5. Analytical Methods

#### 2.5.1. Extracellular Polymeric Substances (EPS)

The concentration of EPS in the water sample was quantitatively calculated by analyzing the content of carbohydrates (soluble polysaccharides) and proteins. Carbohydrates were determined with an anthrone colorimetric assay, and proteins were measured with the modified Lowry method [23,24].

#### 2.5.2. Determination of Relative Molecular Weight Distribution

In this study, a high-performance size exclusion chromatography-UV detector-TOC detector (HPSEC-UV-TOC, with UV detector from Waters USA, and TOC detector from Shimadzu, Tokyo, Japan) was used to determine the molecular weight distribution (MW) of organic matter. The column was TSK-GEL G3000PWXL (Japan TOSOH Co., Ltd., Tokyo, Japan), measuring 7.8 mm × 300 mm, and the material was a methacrylate copolymer. The guard column was made of a TSK-GEL PWXL Guard column measuring 6.0 mm × 40 mm.

#### 2.5.3. Three-Dimensional Excitation-Emission Matrix (EEM) Fluorescence Spectroscopy

A VARIAN Cary Eclipse fluorescence spectrophotometer (Agilent Technologies, Inc., La Jolla, CA, USA) was applied in this study. The excitation source was a xenon lamp, with an excitation wavelength of 200–400 nm, the emission wavelength of 250–550 nm, the excitation slit width of 10 nm, an emission slit width of 2 nm, and a scanning speed of 12,000 nm/min. The fluorescence intensity was reduced with decreasing pH. Therefore, the pH of the water sample was adjusted to about 7.0 before the measurement, and then tested at room temperature (25 °C) using a 1 cm fluorescent cuvette. To eliminate the effects of pure water, the blank experiment was measured with ultrapure water before scanning the sample. The resulting data was processed into contour plots using Origin 8.5 and Surfer 8.0 software (OriginLab, Northampton, MA, USA) to characterize the fluorescence information of the organics in the sample. The fluorescent region boundaries and characteristic substance types are displayed in Table S3 [25,26].

#### 2.5.4. Separation of Hydrophilic and Hydrophobic Components

The DAX and XAD resin separation methods can be used to separate NOM into three components. DAX-8 and XAD-4 resins were adopted to adsorb and elute hydrophobic components (HPO) and transphilic components (TPI). If it was not retained by the above two resins, it was a hydrophilic component (HPI). The water sample was filtered through a 0.45 μm filter and 250 mL was collected for separation. The effluent pH was adjusted to 2.0 with a 5 mol/L HCl, and 50 mL passed through the DAX-8 and XAD-4 ion exchange columns trapped in series at a flow rate of 1.5 to 2.5 mL/min. Then, the two columns were eluted with 0.1 mol/L NaOH each at a flow rate of 0.5 to 1.0 mL/min to obtain the HPO and HPI components, respectively. The organic component that was not adsorbed through the two columns was the HPI component. After adjusting the pH of the HPO, TPI, and HPI solutions to about 7.0, the corresponding DOC, UV_254_ and MW distribution were measured.

## 3. Results and Discussion

### 3.1. Membrane Operating Conditions and Variations in Organic Matter

#### 3.1.1. Effect of Filtration Performance and Variation in Organic Matter Removal

**Figure 1:**
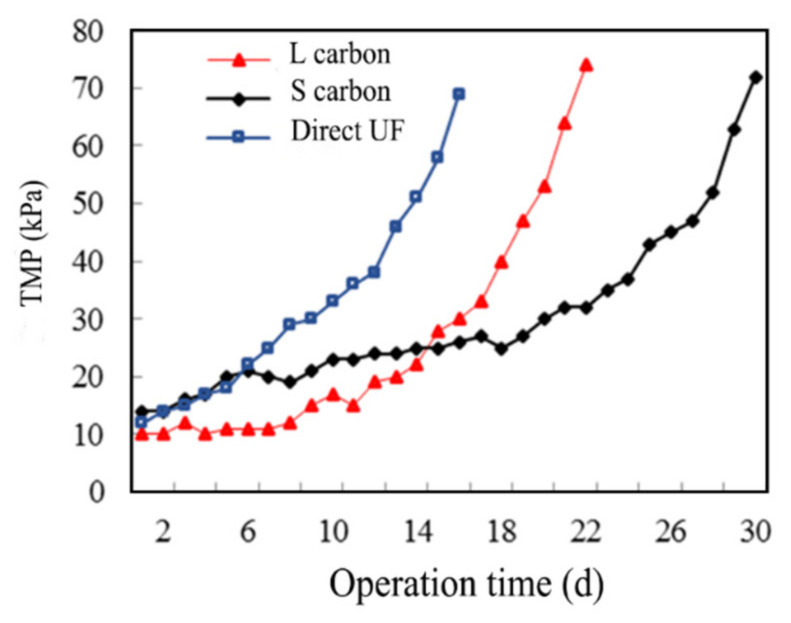
Effects of operating conditions on TMP performance.

The effects of operating conditions (direct UF, S carbon, and L carbon) on the TMP variations are illustrated in Figure 1. It could be observed that the TMP increased slowly at different operating conditions in the initial stage of operation, and the TMP of the direct UF, L-UF (the enhanced reactor with L carbon addition was abbreviated as L-UF), and S-UF (the enhanced reactor with S carbon addition was abbreviated as S-UF) increased rapidly after 12, 16, and 26 days. The direct UF filtration, L-UF, and S-UF conditions lasted 15 d, 22 d, and 30 d, respectively, when serious membrane fouling occurred with the TMP above 70 kPa. Thus, after adding PAC, the operation cycle was prolonged, with the S-UF reactor exhibiting superior performance, which was probably attributed to that PAC could adsorb the organics in the reactor, thus reducing the UF membrane fouling.

Figure S6 provides the removal ratio of DOC and UV_254_ for all three operations. Clearly, after PAC addition, the removal effect of the reactor on DOC was significantly improved compared with direct UF filtration. The DOC removal ratio of direct UF filtration was only about 7%, while the removal ratio of L-UF was 55% and 47% for S-UF after PAC strengthening. Similar to the DOC, the PAC-UF had a much higher UV_254_ removal ratio of above 50%, while the direct UF exhibited a much lower ratio of around 15%. This may be due to that PAC had a large specific surface area and could adsorb some organic matters [27]. At the same time, the PAC concentration in the mixing tank was stable at about 4 g/L, which provided an attachment carrier for the microorganisms. After the initial bioaugmentation, the microorganisms gradually adhered to the surface of the PAC to form a biofilm, which could degrade the organic matter together with the unattached microbial flocs [28].

The removal ratio of the UV_254_ in the reactor with S carbon addition was slightly higher than that with L carbon, which may be ascribed to the following reasons, i.e., the surface of the S carbon had more basic groups, and it was easier to adsorb the organic substances with ultraviolet response; the microorganisms presented in the S-UF mixing tank had a higher removal ratio of organic substances with ultraviolet response [29]. In addition, the average removal ratio of UV_254_ of L-UF and S-UF was higher than that of DOC, which may be due to that microorganisms in the mixing tank were more likely to degrade organic substances with strong absorption of ultraviolet rays containing unsaturated bonds. In addition, the DOC and UV_254_ of the effluent exhibited similar fluctuations with the raw water, extrapolating that the UF membrane itself could also intercept the organic matter, but the effect was greatly affected by the original water content [30].

#### 3.1.2. EPS Variation

**Figure 2:**
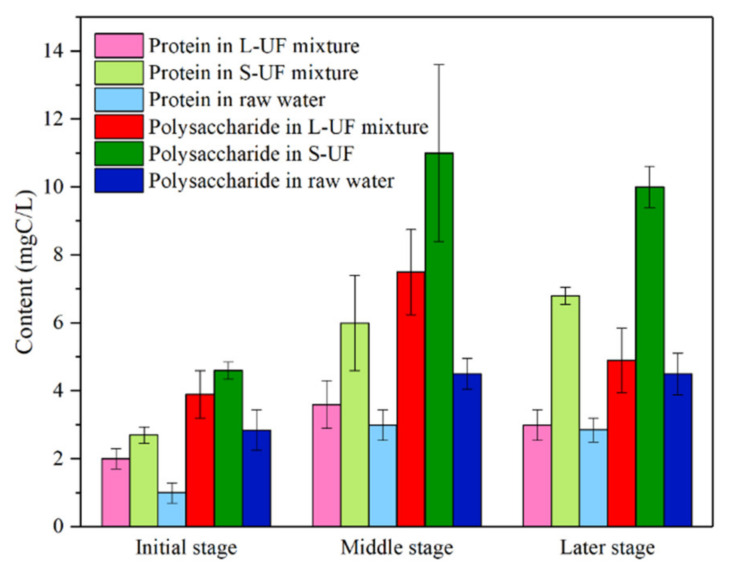
Variation in soluble EPS content in raw water and mixed liquor in L-UF and S-UF reactors.

Figure 2 provides the variation of soluble EPS content in raw water, L-UF, and S-UF mixtures and Table S4 presents the ANOVA statistical analysis using Tukey’s test of the comparison of EPS concentration in terms of both protein and polysaccharide from the raw water, the L-UF and S-UF reactors in the initial, middle and later stages. As shown in Figure 2, the protein content was lower than that of the polysaccharide for the three water qualities. After adding carbon, the protein and polysaccharide content of the mixture were significantly higher than the raw water during the whole operation. It could be deduced that there were active microorganisms in the mixture, and as the operation proceeded, the content of protein and polysaccharide in the mixture of L-UF and S-UF demonstrated a trend of increasing first and then decreasing. The variation of protein and polysaccharide contents in the raw water was smaller in the middle and later stages than in the mixture, surmising that there was an increase or decrease in the proliferation and metabolism of the cultured microorganisms. Meanwhile, the EPS content of the S-UF mixture was higher than that of L-UF, especially in the middle and later stages, indicating that the microbial metabolism in S-UF was more vigorous, which might result from the favored growth of microorganisms in the larger pore volume of the S carbon.

In addition, the increase in the EPS content in the reactor could lead to an increment in the particle size of mixed liquor. Table S5 presents the variation in particle size of the mixed liquor during the operations. It can be observed that the particle size in both the L-UF and S-UF reactors increased when the filtration proceeded. Specifically, the size grading in the S-UF reactor is more significant, toward the right, i.e., a larger particle size (Figure S7). In the middle stage of the operation, the average particle size of the mixed liquor was over 200 µm in S-UF, which was much larger than that in the L-UF. Thus, the EPS in the S-UF was much higher than that in the L-UF (Figure 2).

#### 3.1.3. Molecular Weight (MW) Distribution

**Figure 3:**
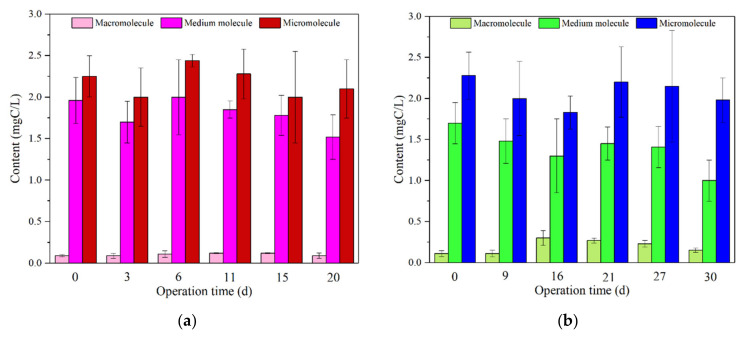

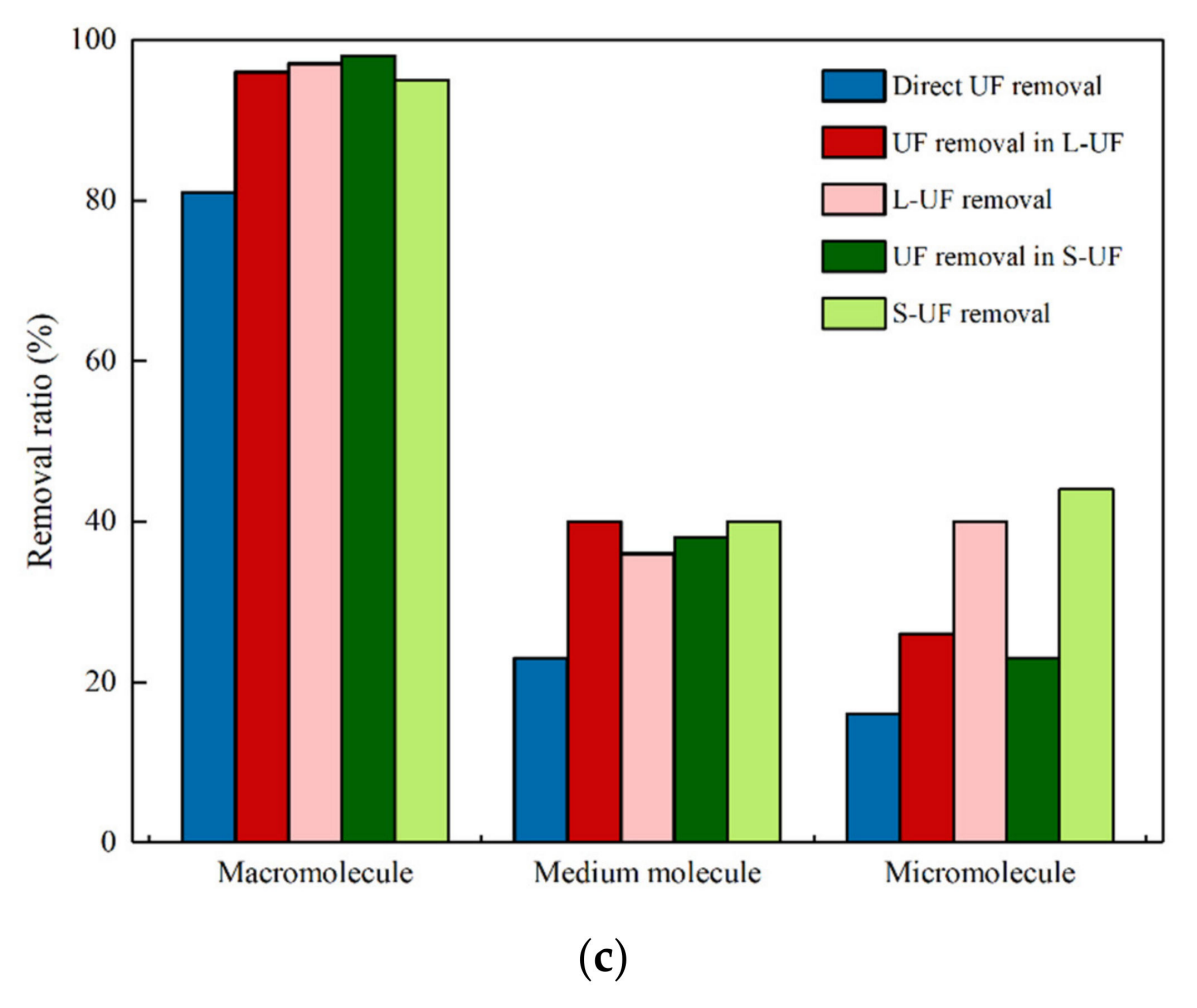
(**a**,**b**) MW variations of the mixed liquor in the L-UF reactor and S-UF reactor, and (**c**) removal ratio of different interval molecular weights.

The molecular weight distribution of the water samples in the raw water, effluent, and mixing tank were examined, and the results are shown in Figure 3. From Figure 3a,b the organic matter in raw water could be divided into three sections, i.e., macromolecule, medium molecule and small molecule. In the initial stage of operation, the peak of macromolecules in the mixture of L-UF and S-UF was very low, while it was very strong for medium and small molecules. Meanwhile, the medium and small molecular peaks of the organic matter in the reactor and effluent reduced as compared with that in the raw water, indicating the content of humic organic matter and small molecular protein in the incubator had a considerable decline. According to the analysis of the nature of the activated carbon, the decrease of L-UF was larger than that of S-UF. This may be due to that the adsorption capacity of L carbon was stronger, and at this time, the growth of L-UF microorganisms was earlier. In the middle of the operation, there was no significant change in L-UF, i.e., the peak of macromolecules was small, and the decrease of small and medium molecules was similar to that in the initial stage. However, the peak of the macromolecules was observed in the mixture of S-UF, with the intensity much larger than that in the raw water. The content of small molecules decreased significantly. This may be attributed to the fact that the microbes in S-UF proliferated and the hypermetabolism was enhanced. A large number of hydrophilic small molecules could be degraded or synthesized into macromolecular organics, but the microbial activity in L-UF showed no significant change. At the end of the operation, the reduction of organic matter in the molecular weight range of the two working conditions significantly decreased, and the removal effect was slightly worse than in the medium and early stages. The reason for the performance may be that the microbial activity decreased with the decline of the microbial organic matter, the degradation ability of the small molecule organic matter was reduced, and some sedimentation occurred.

The peak area of Peakfit was used to calculate the percentage of each molecular weight range. Comparing the molecular weight content of the mixture in the L-UF and S-UF mixing tanks (Figure 3a,b), it could be seen that the content of both macromolecules increased and then decreased. The variation of medium and small molecules was the same, and they were decreasing first, then increasing, and then decreasing. This phenomenon may be attributed to the microbial activity and the fluctuation of raw water quality. The medium molecular content of the S-UF mixture was lower than that of L-UF. The reason may be as follows, i.e., PAC showed an obvious adsorption effect at the initial stage of operation, especially for adsorbing small molecular substances, but the adsorption was quickly weakened, and the amount of carbon added per day was small, relying solely on PAC; the addition of PAC was beneficial to the growth of microorganisms, and in the process, microorganisms selectively consumed a higher proportion of smaller molecular weight organic matter, synthesized, and produced macromolecular substances during metabolism. The separation principle of the UF membrane made it easier to retain large molecules. Therefore, the macromolecular organic matter was accumulated in the mixing tank, and the decrease in the later stage may be due to the influence of the decrease of the microbial metabolic rate and the fluctuation of the raw water quality. It has been reported that PAC particle size significantly affects the growth environment of microorganisms in the system [31,32]. The smaller the PAC particle size, the more obvious the growth of microorganisms in the system was inhibited. As shown in Figures S1–S3, comparing with the L carbon, the S carbon has a larger average particle size and a relatively larger pore volume in 0–5 nm and over 30 nm. The larger particle size and pore volume of S carbon may provide a more favorable growth environment for microorganisms. Therefore, more microbes, more metabolism, and more synthetic macromolecules were produced in the S-UF reactor.

From the removal ratio results, as shown in Figure 3c, the direct UF filtration had a good retention effect on macromolecules, with a removal ratio of over 80%. After adding activated carbon, the removal ratio increased to 96% or even more. UF membranes have the ability to retain medium and small molecules, especially for small molecules (less than 20%). This was owing to that membrane filtration was one of the mechanical actions, which included adsorption, blockage, and mechanical retention [33]. Contaminants with particle size larger than the membrane pore size could be effectively retained [34]. Clearly, the addition of PAC enhanced the removal effect of NOM. Regardless of the carbon added, the total removal ratio of the effluent from large, medium, and small molecules relative to raw water was increased compared with direct UF filtration, and the removal ratio of small molecules was the largest. After being activated by PAC, it exceeded 40%. However, the removal ratio of small molecular substances exhibited a minimum value. This was attributed to the fact that the composition of the mixture after carbon addition had a significant reduction in small molecules, and some weakly polar and biodegradable small molecular organic substances were consumed by the activated carbon adsorption and biological metabolism [35].

#### 3.1.4. Three-Dimensional Fluorescence (EEM) Spectroscopy

**Figure 4:**
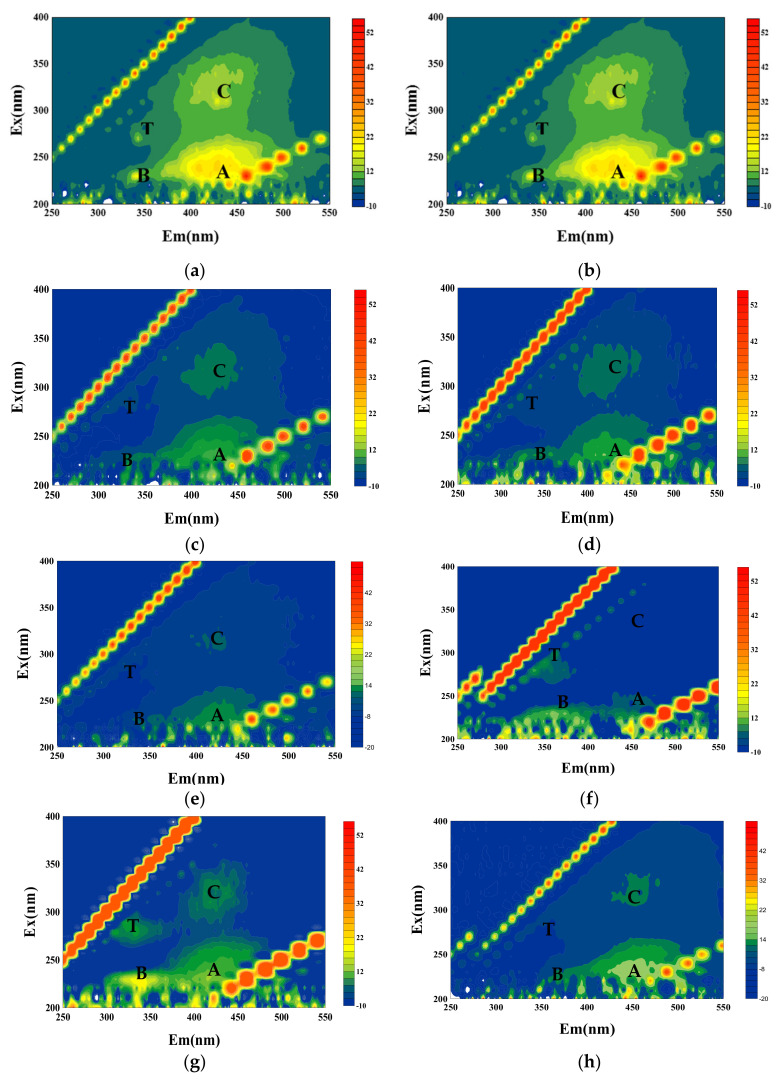
EEM spectra of the water sample of raw water, effluent, and mixing tank under different conditions: (**a**) raw water; (**b**) direct UF filtration, effluent; (**c**) L-UF, initial mixture; (**d**) L-UF, middle and later mixture; (**e**) L-UF, effluent; (**f**) S-UF, initial mixture; (**g**) S-UF, middle and later mixture; and (**h**) S-UF, effluent.

The EEM spectra of raw water, effluent, and mixed liquor in the reactor under different working conditions are shown in Figure 4. There were four peak regions, B, T, A, and C, in the Sanhaowu raw water. The protein and humic areas had an obvious response, but the A and C peaks were strong, and the B and T peaks were weak, indicating that the raw water in the filtration test contained more humic substances and protein substances. However, comparing the effluent of direct UF filtration, it could be observed that all four peaks of the effluent were weakened to varying degrees, and the protein response almost disappeared, but the humic response peak was still evident, indicating that the protein was the main pollution leading to membrane fouling. The UF membrane could hardly remove humic organic matter.

It can be seen from the fluorescence spectra of the L-UF mixture in different stages of operation that the four response peaks of the mixture were significantly weaker than the raw water in the initial stage of operation. Although the change in the middle and later stages was small, the A peak response could be observed, indicating that the addition of the L carbon contributed to the removal of organic matter, especially humic organic matter, by the UF membrane. Similar to L-UF, the peak intensity of the four peaks in the S-UF mixture was weakened at the beginning of the operation, and the C-peak response decreased more, suggesting that the humic organic matter was obviously decreased in the initial stage of the mixed liquid of S-UF. The response peak of proteinaceous material may be caused by the metabolites of raw water and initial microorganisms. In the middle and later stages, enhanced B, T, and C peaks were observed in the mixture. This was mainly attributed to the two sources, i.e., the part introduced by the fluctuation of raw water quality and the metabolism and synthesis of microorganisms. 

### 3.2. Membrane Cleaning Characteristics under Different Conditions

#### 3.2.1. Variation of Membrane Resistance

**Figure 5:**
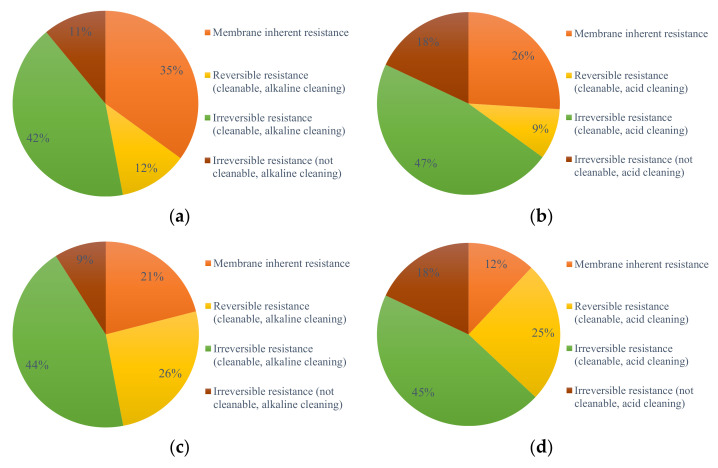

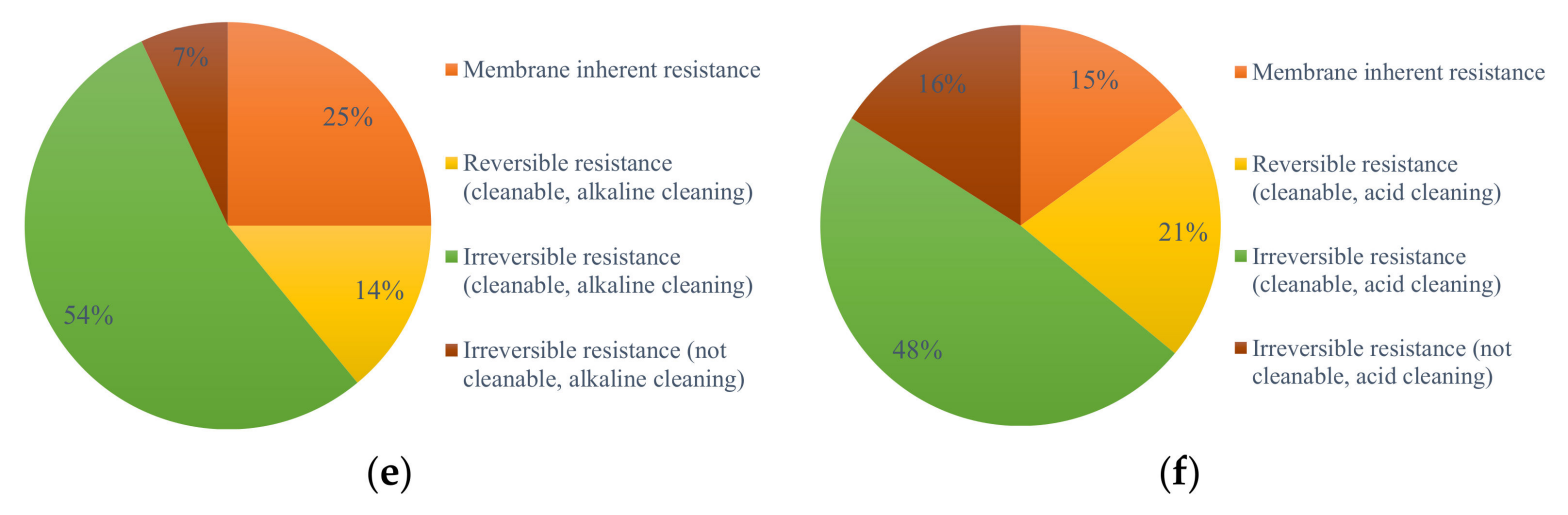
The ratio of the resistance of the membrane after cleaning: (**a**) direct UF filtration, alkaline cleaning; (**b**) direct UF filtration, acid cleaning; (**c**) L-UF, alkaline cleaning; (**d**) L-UF, acid cleaning; (**e**) S-UF, alkaline cleaning; and (**f**) S-UF, acid cleaning.

The membrane modules of the direct UF filtration, L-UF, and S-UF conditions were cleaned, and membrane resistance was observed as a function of TMP change. The resistance distributions are displayed in Figure 5 and Figure S8. Regardless of the TMP of 10 kPa, 15 kPa, or 30 kPa, the order of the resistance of the carbonization condition was irreversible (cleanable, acid cleaning) > irreversible (cleanable, alkaline cleaning) > inherent resistance of the membrane > reversible (alkaline cleaning) > reversible (acid cleaning) > concentration difference resistance. It can be seen that the highest ratio of the inherent resistance of the membrane was 35% of the total filtration resistance in the direct UF filtration, which was significantly higher than the PAC-UF. After the end of the three working conditions, the irreversible resistance ratio reached over 50%, which accounted for the highest proportion of membrane filtration resistance. This phenomenon illustrated that irreversible resistance in the membrane filtration process was the biggest contributor to the membrane resistance growth process. In addition, it was found that the irreversible resistance ratio of acid cleaning was greater than that of alkaline cleaning, which indicated that for irreversible fouling, acid cleaning had greater cleaning efficiency and produced higher membrane recovery. The proportion of reversible resistance was much lower in direct UF filtration than PAC-UF, which could be due to the fact that adding PAC could improve the porous structure of the membrane cake layer, increase reversible fouling, and alleviate irreversible fouling, thus prolonging the filtration duration.

#### 3.2.2. Analysis of Membrane Contaminants

##### Content of Organic Matter in Membrane Elution

The organic matter content of the membrane elution is shown in Table S6. It can be seen that the DOC and UV_254_ values of the alkaline cleaning liquid were the highest in each working condition, followed by acid cleaning, and the lowest physical washing, indicating that the alkaline cleaning could elute more. Comparing the organics in the alkaline cleaning and acid cleaning of the L-UF and S-UF conditions, it could be found that the DOC in the respective alkaline cleaning and acid cleaning was quite different, but the UV_254_ values were similar, indicating that the acid cleaning could elute more easily the substances that respond to ultraviolet light. The DOC in the alkaline cleaning and acid cleaning of L-UF were lower than that of S-UF, but the corresponding UV value was larger than that of S-UF, indicating that in the L-UF cleaning solution, UV responses, such as humic acid and aromatic organic compounds containing carbon-oxygen double bonds, accounted for a higher proportion.

**Figure 6:**
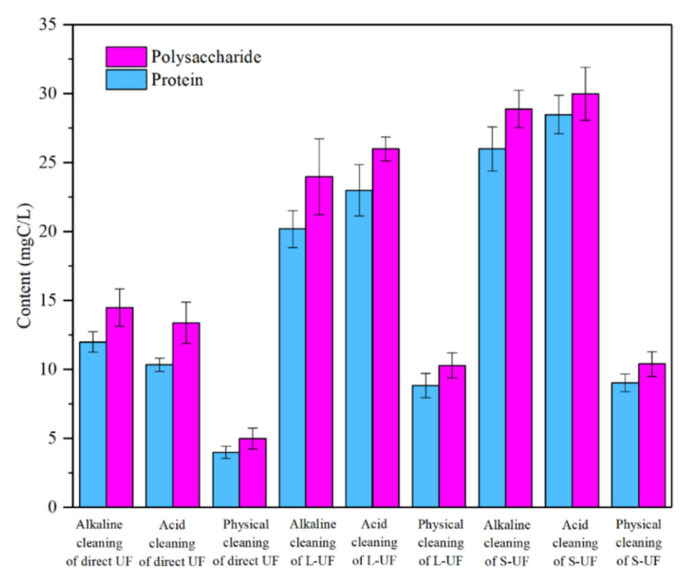
EPS content in membrane elution.

The content of soluble EPS in the cleaning solution is shown in Figure 6, with the ANOVA statistical analysis using Tukey’s test of the comparison of EPS concentration in terms of both protein and polysaccharide from the direct UF, L-UF, and S-UF reactors with the physical, acid, and alkaline cleanings are presented in Table S7. Comparing the EPS concentration in the membrane elution from L-UF and S-UF reactors with that from the direct UF reactor, the three cleaning protocols all behaved significantly differently. While comparing the EPS concentration in the membrane elution from the L-UF reactor with that from the S-UF reactor, the alkaline cleaning exhibited significantly different, and the physical cleaning was not. While the acid cleaning behaved statistically significantly different for the proteins in the membrane elution but did not for polysaccharides. As shown in Figure 6, the content of polysaccharide and protein in the cleaning solution of each working condition was higher than that of the previously tested mixture and raw water, indicating that the polysaccharide and protein contributed greatly to membrane fouling. As depicted in Figure 6, the content of EPS in the direct UF filtration was the lowest, and the highest in the S-UF. Meanwhile, the EPS content of the cleaning solution after acid cleaning was slightly higher than that of the alkaline cleaning, indicating that the acid cleaning was more likely to elute polysaccharide and protein.

##### Molecular Weight Distribution

**Figure 7:**
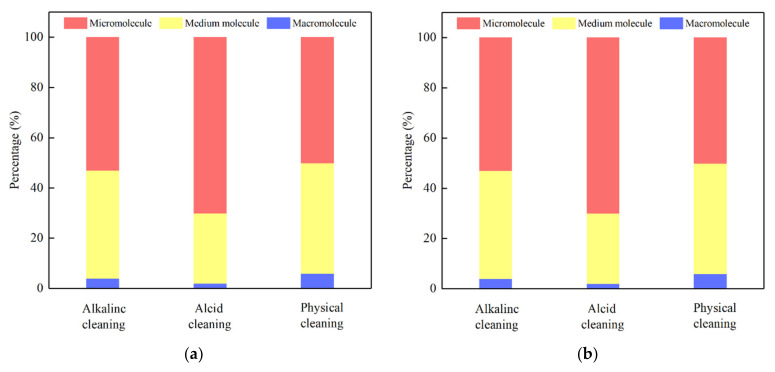

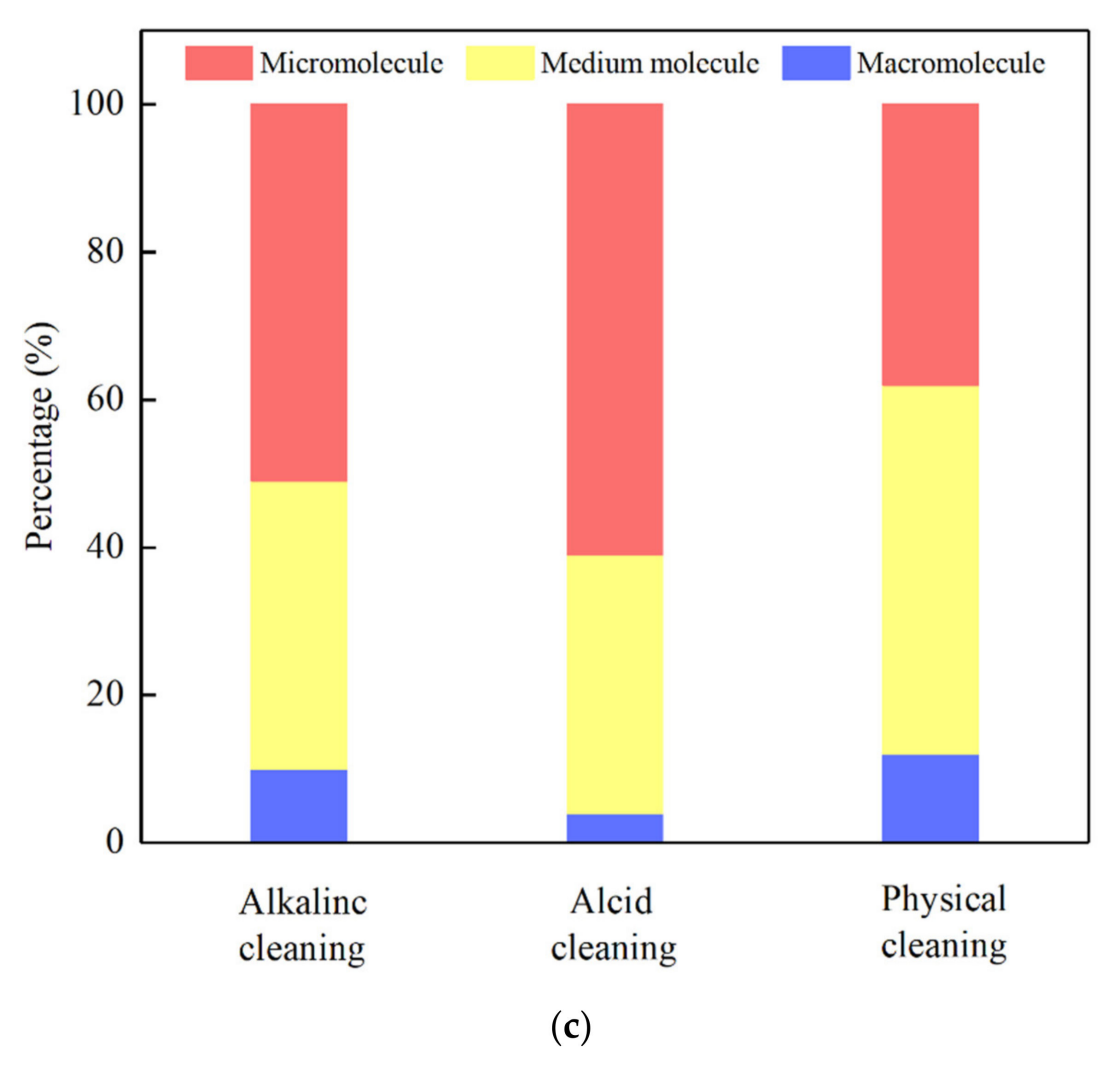
The MW of the membrane elution: (**a**) direct UF; (**b**) L-UF; and (**c**) S-UF.

Figure 7 displays the peak separation analysis of the solution after membrane washing using Peakfit. It could be observed that the direct UF filtration, L-UF, and S-UF had the least proportion of macromolecules in the elution (≤15%), which could be due to the fact that the organic matter in the elution was composed mainly of small and medium molecules.

Among the alkaline cleaning of L-UF, the proportion of medium molecules was the highest, and the other elution had the highest proportion of small molecules, indicating that the small molecules were more likely to cause irreversible fouling of the membrane. In addition, the proportion of small molecules in the acid cleaning solution was the highest among the three cleaning methods, again demonstrating that acid cleaning made it easier to elute small molecular organics [36].

##### Hydrophilic-Hydrophobic Property Analysis of Membrane Elution

**Figure 8:**
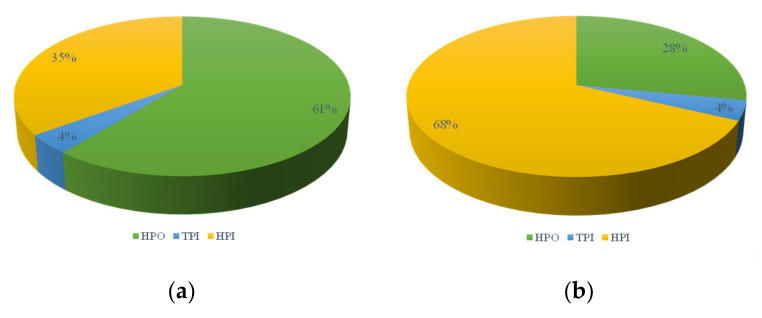

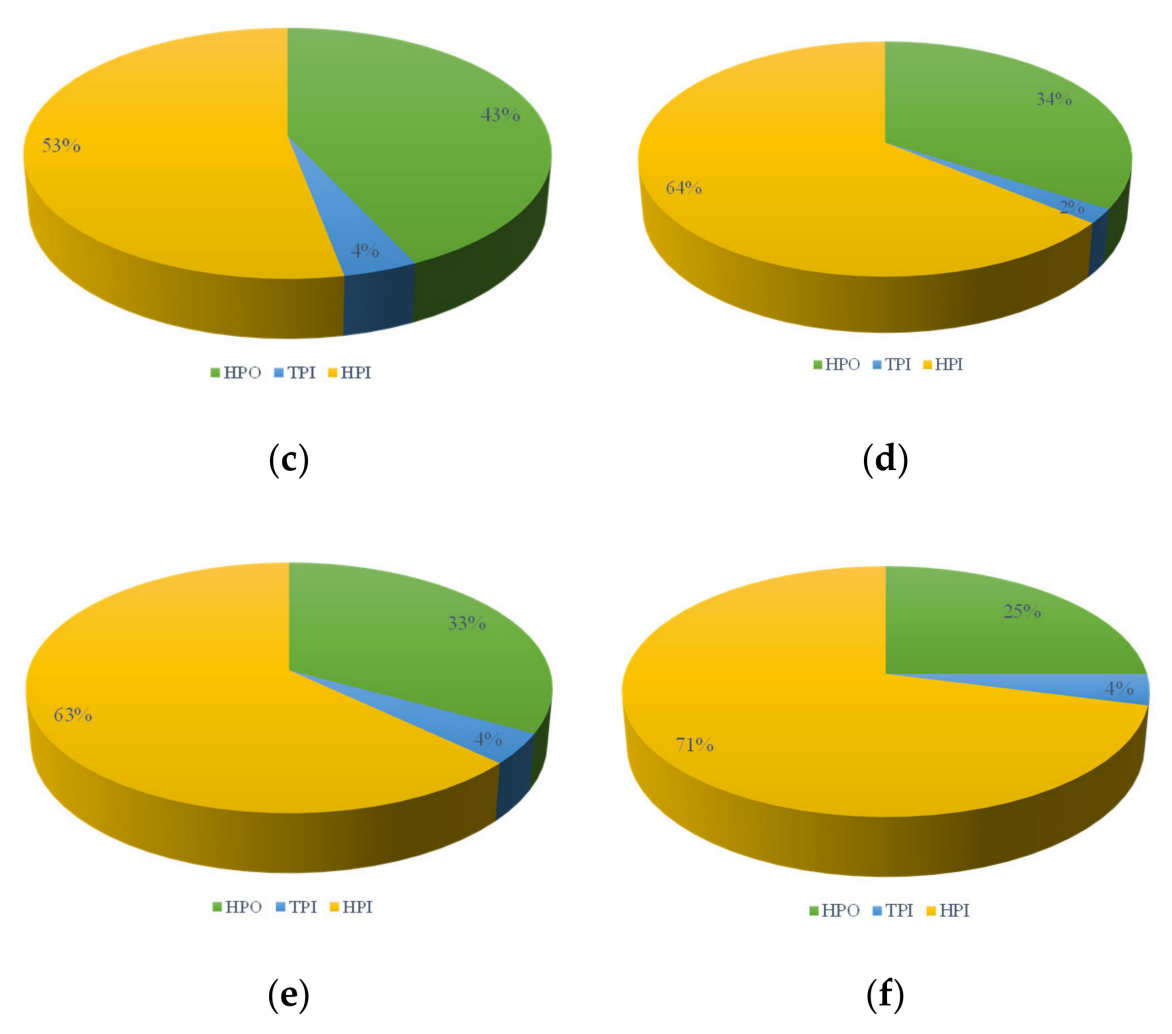
Proportion of hydrophilic and hydrophobic components: (**a**) direct UF filtration, alkaline cleaning; (**b**) direct UF filtration, acid cleaning; (**c**) L-UF, alkaline cleaning; (**d**) L-UF, acid cleaning; (**e**) S-UF, alkaline cleaning; (**f**) S-UF, acid cleaning.

The characteristics of the hydrophilic and hydrophobic separation organics of the membrane elution are shown in Figure 8. Clearly, the main organic substances of the elution were hydrophilic and strong hydrophobic components, and the proportion of weakly hydrophobic components was less than 5%. In the same working condition, the ratio of hydrophilic components was higher in the acid cleaning solution than in the alkaline and physical cleaning, indicating that acid cleaning was more prone to elute the hydrophilic components. The DOC of the alkaline cleaning liquid in the direct UF filtration condition was mainly composed of strong hydrophobic organic matter. The strong hydrophobic content of L-UF in the alkaline cleaning solution (43%) was higher than that of S-UF (33%), indicating that the strong hydrophobic matter had a greater influence on membrane fouling in L-UF. The content of hydrophilic substances in S-UF was the highest, no matter with the alkaline cleaning or the acid cleaning, indicating that hydrophilic organic compounds had a greater impact on membrane fouling in S-UF.

### 3.3. Mechanisms of Different PACs’ Effect on Membrane Filtration

**Figure 9:**
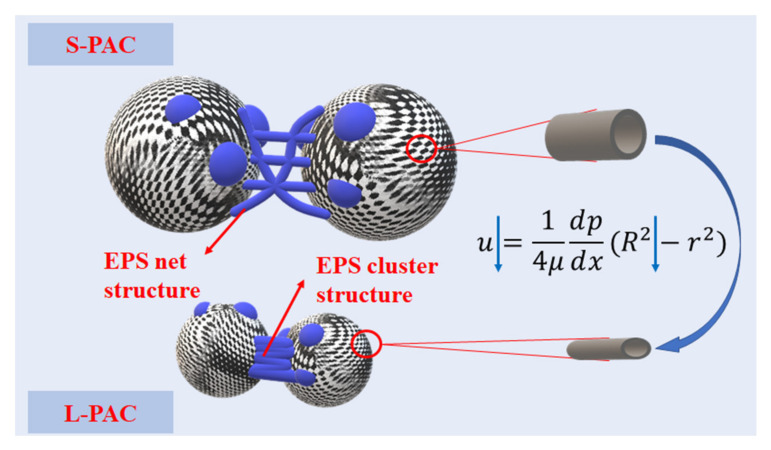
Mechanism of PAC addition on the UF filtration process.

Compared with the L carbon with the smaller particle size and the lower BET, the S carbon with the larger particle size and the higher BET exhibited superior performance in prolonging the UF filtration process, which suggests that the membrane fouling could be better controlled. Since the small PAC particle size could inhibit the growth of microorganisms in the mixed liquor, the S carbon with a larger particle size and pore volume (d > 30.0 nm) provided a more favorable growth environment for microorganisms and covered more EPS on their surface. Therefore, the particle size of the S carbon mixed liquor also increased as the filtration process proceeded. At the end of the filtration, the S carbon cake layer that formed on the UF membrane surface and contained the highest quantity of EPS (i.e., hydrophilic substances, polysaccharide, and protein) could alleviate irreversible fouling effectively. Thus, particle size and pore structure are the two key parameters that should be considered for the PAC addition during the UF filtration process, which can also be analyzed with the Hagen–Poiseuille equation, as shown in Figure 9.

## 4. Conclusions

In this study, the water purification effect and membrane fouling mechanism of two PAC (L carbon and S carbon)-enhanced PVDF UF membranes on micro-polluted water filtration were investigated. The results indicated that adding PAC could effectively enhance the removal ratio of large, medium, and small molecules on the membrane, and played an active role in the water purification effect and membrane fouling reduction. The S carbon with the larger particle size and pore volume exhibited superior performance in controlling membrane fouling. Particle size and pore structure are the two key parameters that are essential for the PAC-UF process. The composition of the carbon-added mixture was analyzed, and the results showed that with the increase of the operation time, the EPS content of the S-UF mixture increased significantly. The molecular weight distribution results showed that the content of macromolecules in the L-UF and S-UF mixtures increased first and then decreased as the S-UF increased more. With the two PACs, the total removal ratio of large, medium, and small molecular organic matter was increased as compared with direct UF filtration, with the removal ratio of small molecule the largest. After acid cleaning and alkaline cleaning, irreversible fouling played the most important role in the growth of membrane resistance. In addition, the irreversible resistance ratio of acid cleaning was greater than that of alkaline cleaning, indicating that for irreversible fouling, acid cleaning could produce greater cleaning efficiency and membrane recovery. The reversible resistance of direct UF filtration was much lower than PAC-UF, demonstrating that adding activated carbon could improve the structure of the membrane cake layer and increase reversible fouling, thus relieving irreversible fouling to some extent and prolonging the running duration. Finally, through the analysis of the solution after membrane cleaning, it was found that the elution was mainly composed of small and medium molecular organic substances, and polysaccharides were more likely to contribute to membrane fouling than proteins. Alkaline cleaning mainly eluted large, medium, and small molecules of hydrophilic and hydrophobic organic matter, while acid cleaning mainly eluted small molecules of hydrophilic organic matter.

## Data Availability

The datasets analyzed during the current study are available from the corresponding author on reasonable request.

## Supplementary Materials

The following supporting information can be downloaded at: https://www.mdpi.com/article/10.3390/membranes12050487/s1, Figure S1: Particle size distribution of L carbon and S carbon; Figure S2: The pore structure analysis of L carbon and S carbon; Figure S3: The specific surface area analysis of the PAC pores; Table S1: The size distribution of the two different activated carbons (µm); Table S2: Surface oxygen-containing functional group of L carbon and S carbon; Figure S4: Schematic diagram of the experimental setup, (a) direct UF filtration; (b) PAC-UF filtration; Figure S5: Cleaning process diagram; Table S3: Fluorescent region boundaries and characteristic substance types; Figure S6: Removal effects of DOC, UV_254_ at different operating conditions (direct UF filtration, L-UF and S-UF filtration); Table S4: ANOVA statistical analysis of the comparison of EPS concentrations in the raw water, the L-UF and S-UF reactors in the initial, middle and later stages using Tukey’s test; Table S5: The particle size variation of the mixed liquor during operations (µm); Figure S7: The particle size variation of mixed liquor during operations; Table S6: Organic content in the membrane elution; Table S7: ANOVA statistical analysis of the comparison of EPS in the membrane elution from direct UF, L-UF and S-UF reactors with physical, acid and alkaline cleanings using Tukey’s test; Figure S8: The membrane resistance in different operations.
