# Supplementary material for: PAC-UF Process Improving Surface Water Treatment: PAC Effects and Membrane Fouling Mechanism
Source: Membranes (Basel). 2022 Apr 29;12(5):487. doi: 10.3390/membranes12050487 (PMC9143739; doi:10.3390/membranes12050487)
Supplement: Supplementary file 1 [file membranes-12-00487-s001.zip › membranes-1676924-supplementary.pdf]

Supplementary materials

PAC-UF Process Improving Surface Water Treatment: PAC Effects and Membrane Fouling Mechanism

Tian Li [1,2,3], Hongjian Yu [2], Jing Tian [2], Junxia Liu [4,*], Tonghao Yuan [2], Shaoze Xiao [2], Huaqiang Chu [1,2,3,*] and Bingzhi Dong [1,2,3]

1 Key Laboratory of Yangtze River Water Environment, Ministry of Education, College of Environmental Science and Engineering, Tongji University, Shanghai 200092, China; litian001@tongji.edu.cn (T.L.); dbz77@tongji.edu.cn (B.D.)
2 State Key Laboratory of Pollution Control and Resource Reuse, College of Environmental Science and Engineering, Tongji University, Shanghai 200092, China; 1853855@tongji.edu.cn (H.Y.); taylortian@tongji.edu.cn (J.T.); 1952483@tongji.edu.cn (T.Y.); shaoze_xiao@163.com (S.X.)
3 Shanghai Institute of Pollution Control and Ecological Security, Tongji University, Shanghai 200092, China
4 School of Civil and Transportation Engineering, Guangdong University of Technology, Guangzhou 510006, China
* Correspondence: whjunxia@163.com (J.L.); chuhuaqiang@tongji.edu.cn (H.C.); Tel.: +86-20-39322515 (J.L.); +86-21-65985811 (H.C.)

1. Particle size distribution of L carbon and S carbon

The particle size distribution of L carbon and S carbon is shown in Figure S1. The average size of L carbon was smaller than S carbon. The pore volume of L carbon was larger than S carbon (Figure S2), especially in the pore size range of 0-15 nm. The specific surface area of both activated carbons was composed of micropore (d < 2.0 nm) and primary mesopore (d < 5.0 nm). The specific surface area of L carbon was much larger than S carbon (Figure S3), for the former 1632 m^2/g and the latter 620 m^2/g, respectively. L carbon had a pore peak volume in the size of 0-3 nm, while S carbon had a relatively larger pore volume in 0-5 nm and over 30 nm.

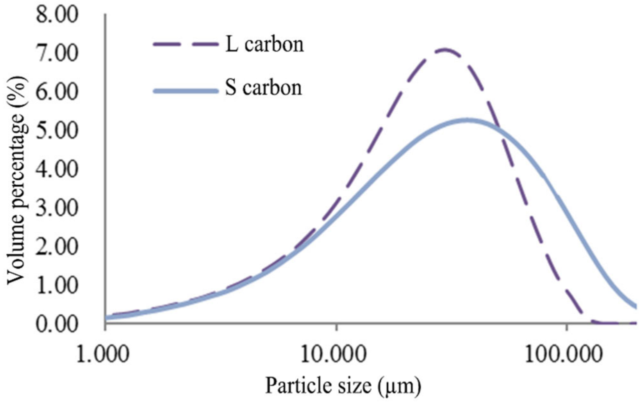

Figure S1. Particle size distribution of L carbon and S carbon.

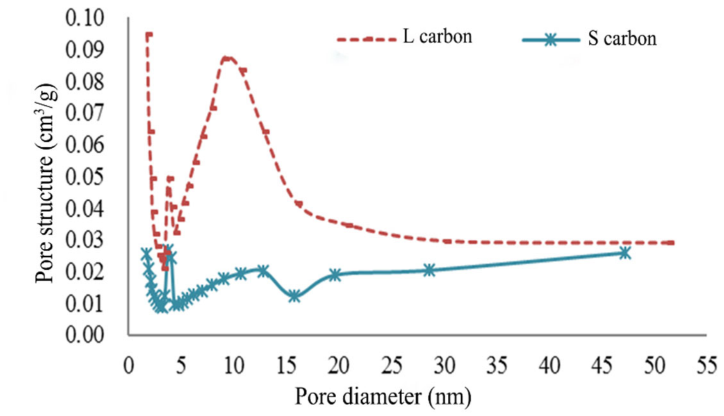

Figure S2. The pore structure analysis of L carbon and S carbon.

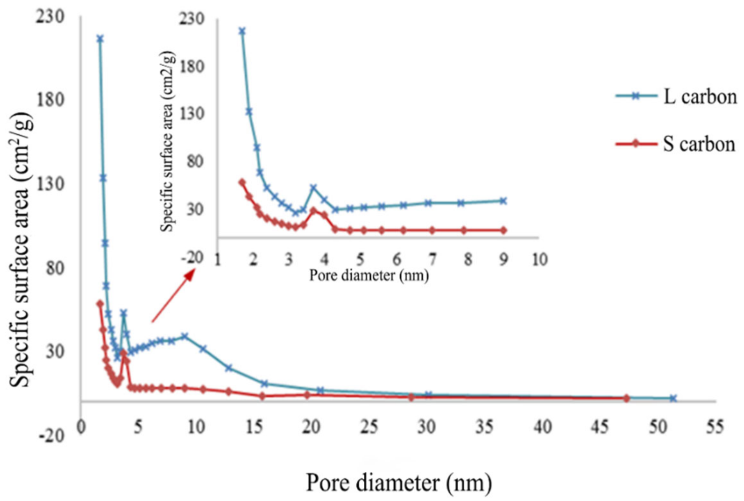

Figure S3. The specific surface area analysis of the PAC pores.

The two carbons had similar particle size distribution, mainly in the range of 10-100 μm (Table S1). D(4, 3) represents the average particle size in volume, and D(3, 2) the average particle size in surface area. D(10), D(50), and D(90) are the equivalent diameters of 10%, 50% and 90% volume in the accumulation distribution curve. It can be seen that the L carbon had a smaller diameter than S carbon. The content of acidic oxygen-containing functional groups on the surface of L carbon was higher while the content of basic oxygen-containing functional groups on the surface of S carbon was larger (Table S2).

Table S1. The size distribution of the two different activated carbons (μm).

Type of PAC	D(3, 2)	D(4, 3)	D(10)	D(50)	D(90)
L carbon	21.63	18.93	12.51	22.03	50.73
S carbon	31.78	21.51	16.02	26.71	71.39

Table S2. Surface oxygen-containing functional group of L carbon and S carbon.

Type of PAC	Surface oxygen-containing functional group (m mol/g)		
	Carboxyl group	Lactones group	Basic group
L carbon	1.53	1.28	0.26
S carbon	1.34	0.77	0.99

2. Experimental setup and cleaning process

The diagram of the experimental setup is shown in Figure S4. Both the direct UF filtration and the PAC-UF setups included the raw water tank, the reactor and the clean water tank. In the direct UF filtration setup, the hollow fiber membrane module was immersed in the reactor, while in the PAC-UF setup, the PAC was suspended in the reactor where the membrane module was also submerged inside.

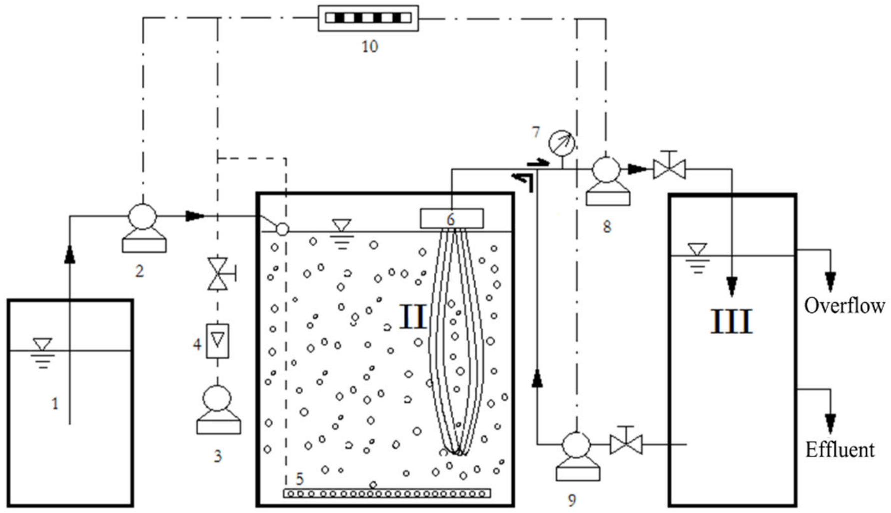

(**a**) Reactor A

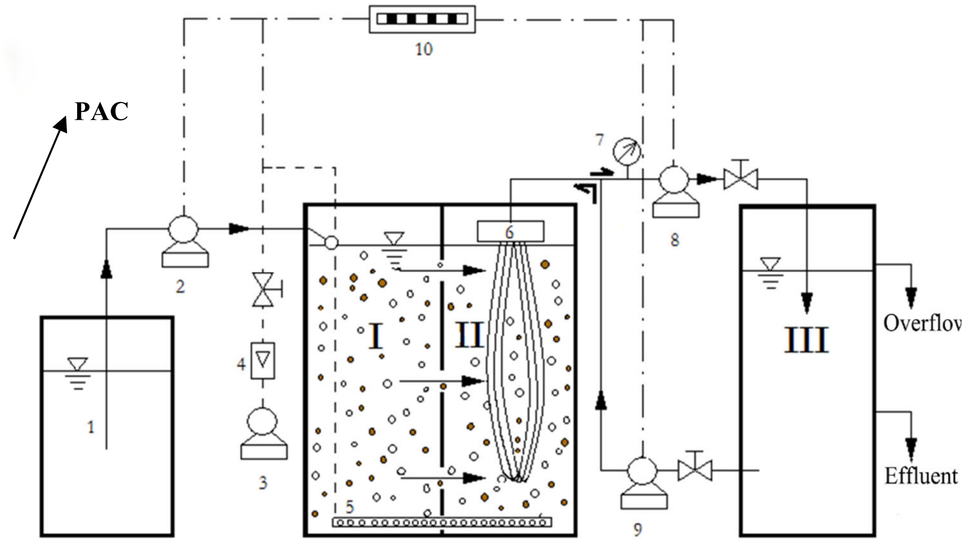

(**b**) Reactor B

Figure S4. Schematic diagram of the experimental setup, (a) direct UF filtration; (b) PAC-UF filtration.

(Notation: (1) raw water tank; (2) feed pump; (3) air pump; (4) air flow meter; (5) air flow meter; (6) ultrafiltration (UF) membrane module; (7) vacuum pressure gauge; (8) vacuum suction pump; (9) backwash pump; (10) electric control box; I: activated carbon mixing tank; II: immersion ultrafiltration (UF) membrane tank; and III: clean water tank.)

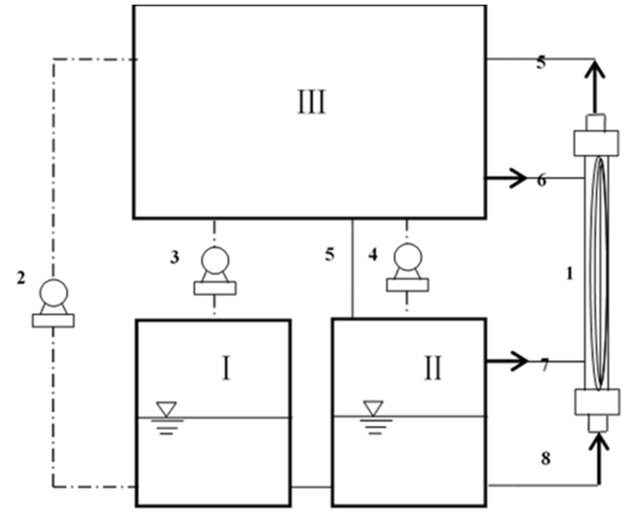

Figure S5. Cleaning process diagram.

(Notation: (1) contamination membrane module; (2) feed water pump; (3) outlet pump; (4) backflush pump; (5) outlet pipe; (6) cross-flow flushing pipe; (7) inlet pipe; (8) recoil pipe; (I) cleaning tank (left); (II) cleaning tank (right); and (III) PLC control box.)

3. Determination of relative molecular weight distribution

The temperature of the detector and column was 40 °C and the ultraviolet detection wavelength was 254 nm. The mobile phase composition included 0.03 mol/L NaOH, 0.05 mol/L KH_2PO_4 and 0.02 mol/L Na_2SO_4. Ultrasonic water was applied to remove the bubbles in the solution before use to avoid damage to the column or the instrument. The flow rate of the mobile phase was 0.50 mL/min.

Sodium polystyrene sulfonate (PSS) having molecular weights of 0.21 kDa, 1.4 kDa, 3.6 kDa, 4.3 kDa, 6.8 kDa, 15.4 kDa, and 31 kDa was used as a standard. The water sample was filtered through a 0.45 μm filter and adjusted to a pH of 5-6. The injection volume was 100 μL and the measurement duration was 35 min.

The gel chromatogram of the tested water samples was analyzed using the software PeakFit v4.12 (a non-linear curve fitting software), the overlapping hidden peaks were separated, and the peak area S_i of different molecular weight intervals was calculated. Then the total area can be computed with $S_T = \sum S_i$. According to this, the percentage of content in a certain molecular weight range could be obtained.

Table S3. Fluorescent region boundaries and characteristic substance types.

Regions	Ex/nm	Em/nm	Main organic types
Region B	220-250	280-380	Aromatic protein, Tyrosine
Region T	250-370	280-380	Soluble microbial products, Tryptophan
Region A	230-280	380-500	Fulvic acid
Region C	280-400	380-500	Humic acid

4. Filtration performance of direct UF and PAC-UF process

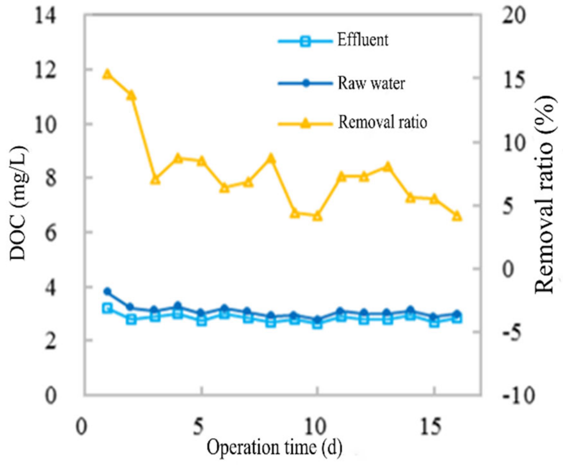

(a)

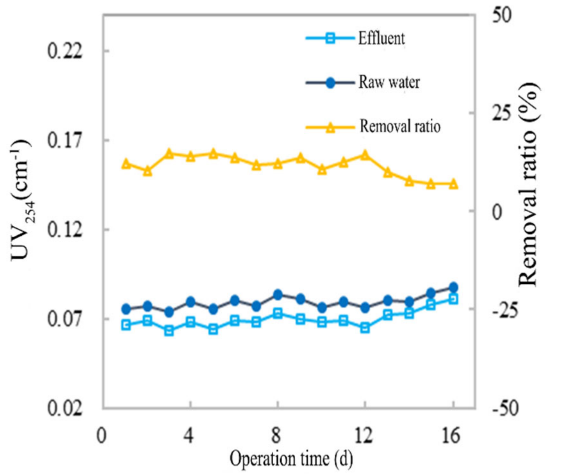

(b)

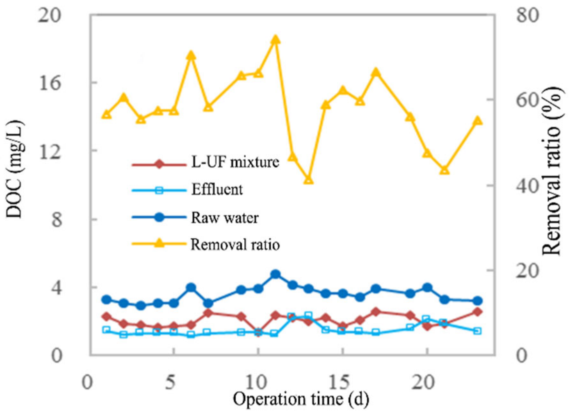

(c)

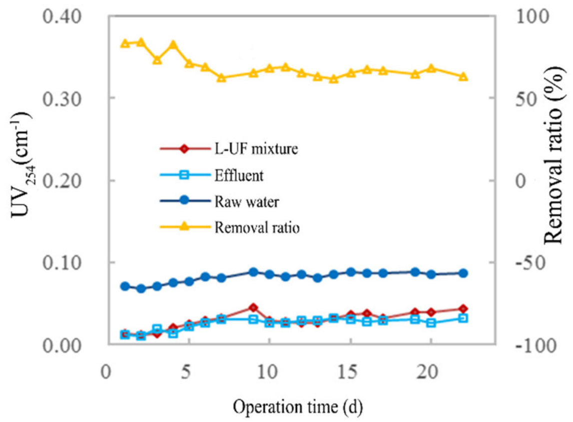

(d)

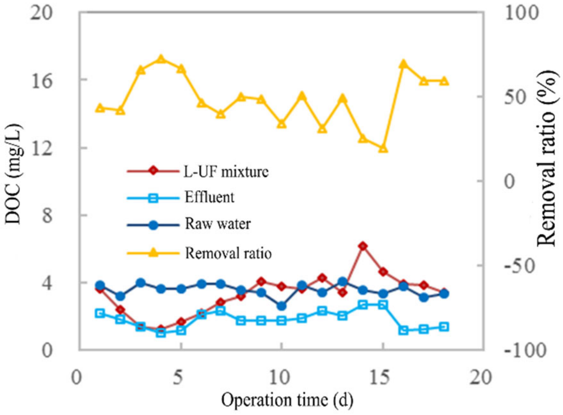

(e)

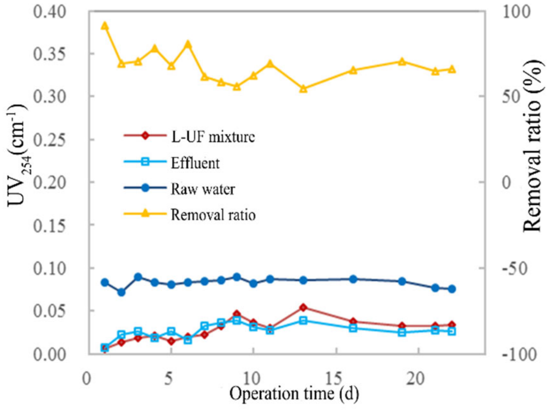

(f)

Figure S6. Removal effects of DOC, UV_{254} at different operating conditions (direct UF filtration, L-UF and S-UF filtration).

(a) direct UF filtration, removal ratio of DOC; (b) direct UF filtration, removal ratio of UV_{254}, (c) L-UF, removal ratio of DOC; (d) L-UF, removal ratio of UV_{254}; (e) S-UF, removal ratio of DOC; and (f) S-UF, removal ratio of UV_{254}.

5. Statistical analysis of the comparison of EPS concentrations in terms of proteins and polysaccharides in the raw water, the L-UF and S-UF reactors in the initial, middle and later stages

Table S4. ANOVA statistical analysis of the comparison of EPS concentrations in the raw water, the L-UF and S-UF reactors in the initial, middle and later stages using Tukey's test.

Stages [a]	Proteins			Polysaccharides		
	Prob	Alpha	Sig [b]	Prob	Alpha	Sig [b]
Initial-S-UF vs. Initial-L-UF	8.55E-01	0.05	0	9.97E-01	0.05	0
Initial-Raw vs. Initial-L-UF	5.48E-01	0.05	0	9.47E-01	0.05	0
Initial-Raw vs. Initial-S-UF	5.10E-02	0.05	0	6.04E-01	0.05	0
Middle-L-UF vs. Initial-L-UF	6.45E-02	0.05	0	1.82E-02	0.05	1
Middle-S-UF vs. Initial-S-UF	5.40E-05	0.05	1	4.85E-05	0.05	1
Middle-S-UF vs. Middle-L-UF	2.39E-03	0.05	1	3.55E-02	0.05	1
Middle-Raw vs. Initial-Raw	1.47E-02	0.05	1	6.70E-01	0.05	0
Middle-Raw vs. Middle-L-UF	9.24E-01	0.05	0	6.32E-02	0.05	0
Middle-Raw vs. Middle-S-UF	1.79E-04	0.05	1	3.94E-05	0.05	1
Later-L-UF vs. Initial-L-UF	5.88E-01	0.05	0	9.72E-01	0.05	0
Later-L-UF vs. Middle-L-UF	8.77E-01	0.05	0	1.43E-01	0.05	0
Later-S-UF vs. Initial-S-UF	4.15E-06	0.05	1	4.17E-04	0.05	1
Later-S-UF vs. Middle-S-UF	8.77E-01	0.05	0	9.66E-01	0.05	0
Later-S-UF vs. Later-L-UF	9.75E-06	0.05	1	8.14E-04	0.05	1
Later-Raw vs. Initial-Raw	3.11E-02	0.05	1	6.04E-01	0.05	0
Later-Raw vs. Middle-Raw	1.00E+00	0.05	0	1.00E+00	0.05	0
Later-Raw vs. Later-L-UF	1.00E+00	0.05	0	1.00E+00	0.05	0
Later-Raw vs. Later-S-UF	6.41E-06	0.05	1	4.17E-04	0.05	1

Notes:. [a] Initial-, Middle-, and Later- represent Initial stage, Middle stage and Later stage, respectively. [b] Sig with the value of 1 represents that the comparison is statistically significantly different at $p < 0.05$ level, while Sig with the value of 0 indicates the comparison is not statistically significantly different at $p < 0.05$ level.

6. The particle size variation of the mixed liquor during operations.

Table S5. The particle size variation of the mixed liquor during operations (μm).

Type of PAC	Time (d)	D(3, 2)	D(4, 3)	D(10)	D(50)	D(90)
S carbon	0	31.1	21.9	16.1	26.2	71.1
	3	19.4	39.3	10.2	29.2	82.9
	6	23.3	49.9	11.7	36.1	124.4
	9	31.1	50.5	17.3	44.9	146.3
	16	31.4	55.2	17.2	59.8	201.2
	21	30.6	66.4	16.0	40.4	137.2
	24	19.5	39.7	10.71	33.8	111.5
L carbon	0	22.6	20.7	13.0	24.3	53.8
	3	24.5	30.2	13.9	32	59.1
	9	20.1	33.8	11.2	26.4	63.6
	12	19.2	30.1	10.9	34.8	65.1
	15	17.7	27.3	10.2	25.9	58.4
	18	18.8	29.5	10.6	24.7	53.7

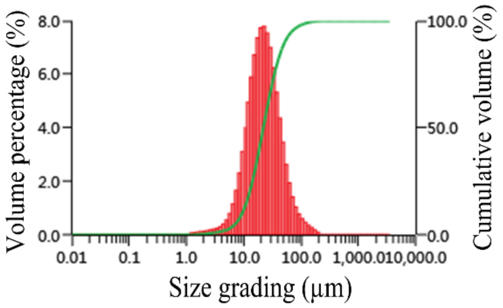

(**a**) L-UF, starting

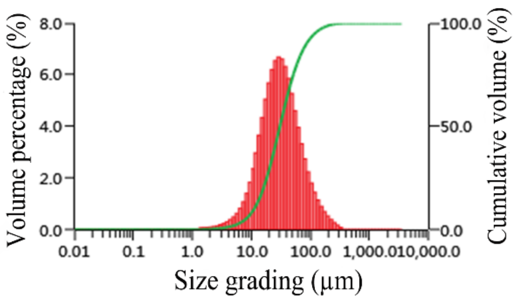

(**b**) S-UF, starting

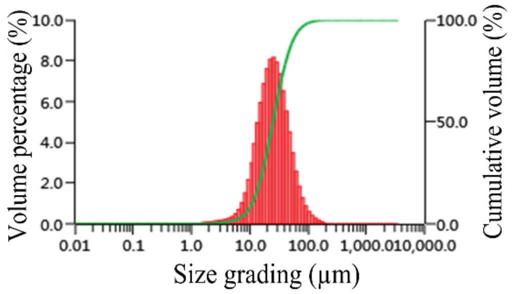

(**c**) L-UF, initial stage

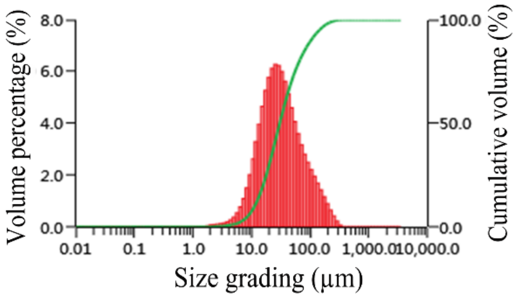

(**d**) S-UF, initial stage

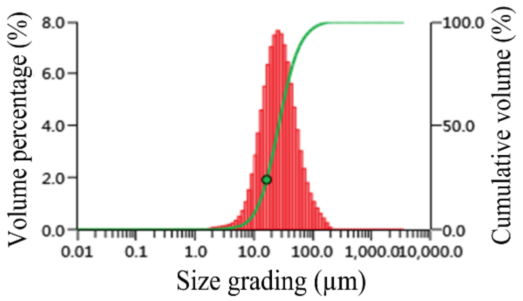

(**e**) L-UF, middle stage

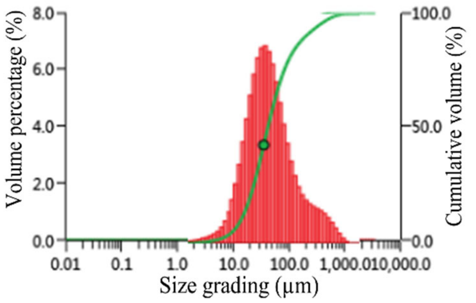

(**f**) S-UF, middle stage

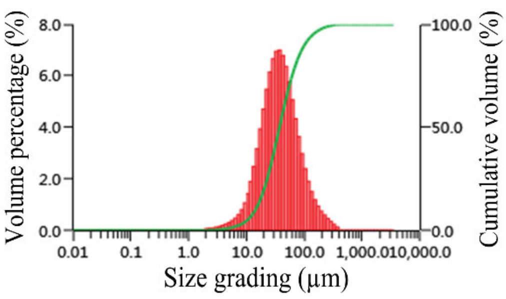

(**g**) L-UF, later stage

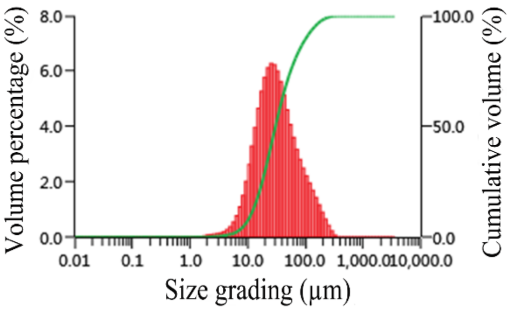

(**h**) S-UF, later stage

Figure S7. The particle size variation of mixed liquor during operations.

7. The organic concentrations in the membrane elution

Table S6. Organic content in the membrane elution.

Conditions	Washing solution	DOC (mg/L)	UV_{254} (cm^{-1})
	Alkaline cleaning	20.86	0.104
L – UF	Acid cleaning	16.85	0.098
	Physical cleaning	5.022	0.013
	Alkaline cleaning	31.49	0.102
S – UF	Acid cleaning	24.35	0.095
	Physical cleaning	3.801	0.008
	Alkaline cleaning	14.66	0.058
Direct UF	Acid cleaning	8.230	0.048
	Physical cleaning	3.020	0.006

8. Statistical analysis of the comparison of EPS concentrations in terms of proteins and polysaccharides in the membrane elution from direct UF, L-UF and S-UF react with the physical, acid and alkaline cleanings

Table S7. ANOVA statistical analysis of the comparison of EPS in the membrane elution from direct UF, L-UF and S-UF reactors with physical, acid and alkaline cleanings using Tukey's test.

Factors [a]	Proteins			Polysaccharides		
	Prob	Alpha	Sig [b]	Prob	Alpha	Sig
Acid-UF vs. Alkaline-UF	6.39E-01	0.05	0	9.95E-01	0.05	0
Physical-UF vs. Alkaline-UF	2.66E-06	0.05	1	1.15E-05	0.05	1
Physical-UF vs. Acid-UF	7.90E-05	0.05	1	5.22E-05	0.05	1
Alkaline-L-UF vs. Alkaline-UF	2.09E-06	0.05	1	1.27E-05	0.05	1
Acid-L-UF vs. Acid-UF	1.12E-07	0.05	1	2.33E-07	0.05	1
Acid-L-UF vs. Alkaline-L-UF	1.35E-01	0.05	0	7.12E-01	0.05	0
Physical-L-UF vs. Physical-UF	1.92E-03	0.05	1	6.89E-03	0.05	1
Physical-L-UF vs. Alkaline-L-UF	9.48E-08	0.05	1	1.42E-07	0.05	1
Physical-L-UF vs. Acid-L-UF	3.82E-07	0.05	1	9.16E-08	0.05	1
Alkaline-S-UF vs. Alkaline-UF	3.77E-07	0.05	1	1.09E-07	0.05	1
Alkaline-S-UF vs. Alkaline-L-UF	1.81E-04	0.05	1	2.01E-02	0.05	1
Acid-S-UF vs. Acid-UF	2.13E-08	0.05	1	1.12E-07	0.05	1
Acid-S-UF vs. Acid-L-UF	3.93E-04	0.05	1	9.96E-02	0.05	0
Acid-S-UF vs. Alkaline-S-UF	2.59E-01	0.05	0	9.88E-01	0.05	0
Physical-S-UF vs. Physical-UF	1.20E-03	0.05	1	9.14E-03	0.05	1
Physical-S-UF vs. Physical-L-UF	1.00E+00	0.05	0	1.00E+00	0.05	0
Physical-S-UF vs. Alkaline-S-UF	2.15E-08	0.05	1	3.78E-07	0.05	1
Physical-S-UF vs. Acid-S-UF	0.00E+00	0.05	1	9.11E-07	0.05	1

Notes: [a] Acid-, Alkaline-, and Physical- represent Acid cleaning, Alkaline cleaning, and Physica cleaning, respectively. [b] Sig with the value of 1 represents that the comparison is statistically

significantly different at p < 0.05 level, while Sig with the value of 0 indicates the comparison is not statistically significantly different at p < 0.05 level.

9. The membrane resistance in different operations

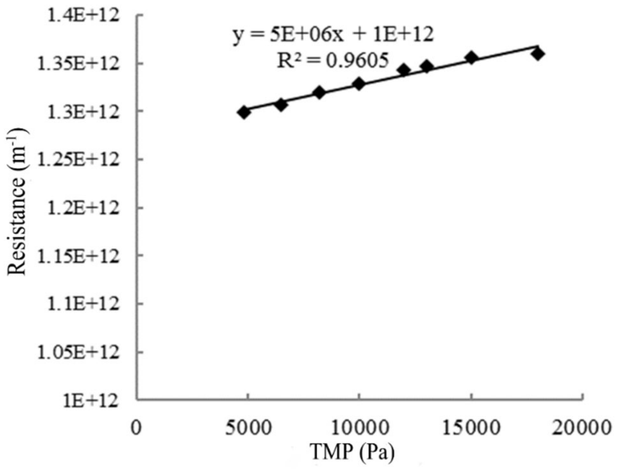

(**a**) Direct UF

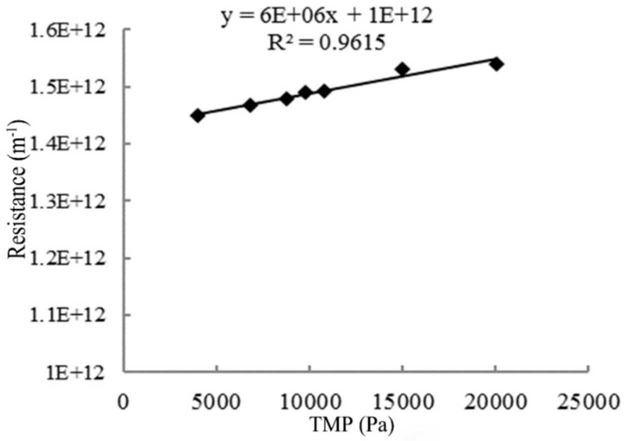

(**b**) L-UF

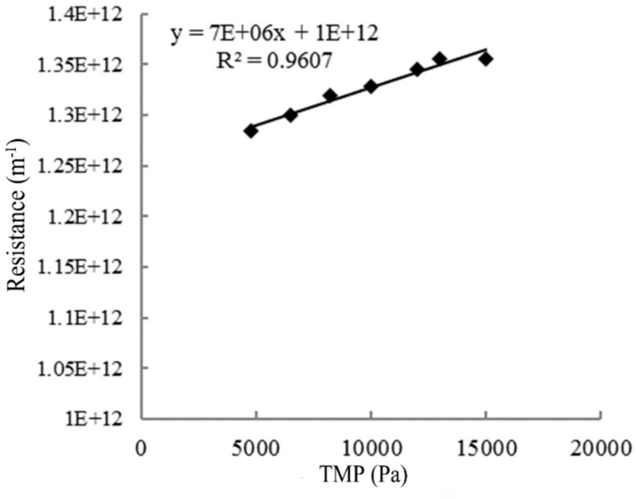

(**c**) S-UF

Figure S8. The membrane resistance in different operations.
